# Undergraduate medical student perceptions and use of Evidence Based Medicine: A qualitative study

**DOI:** 10.1186/1472-6920-10-58

**Published:** 2010-08-19

**Authors:** Dragan Ilic, Kristian Forbes

**Affiliations:** 1 Monash Institute of Health Services Research, School of Public Health & Preventive Medicine, Monash University, Australia

## Abstract

**Background:**

Many medical schools teach the principles of Evidence Based Medicine (EBM) as a subject within their medical curriculum. Few studies have explored the barriers and enablers that students experience when studying medicine and attempting to integrate EBM in their clinical experience. The aim of this study was to identify undergraduate medical student perceptions of EBM, including their current use of its principles as students and perceived future use as clinicians.

**Methods:**

Third year medical students were recruited via email to participate in focus group discussions. Four focus groups were conducted separately across four hospital sites. All focus groups were conducted by the same facilitator. All discussions were transcribed verbatim, and analysed independently by the two authors according to the principles of thematic analysis.

**Results:**

Focus group discussions were conducted with 23 third-year medical students, representing three metropolitan and one rural hospital sites. Five key themes emerged from the analysis of the transcripts: (1) Rationale and observed use of EBM in practice, (2) Current use of EBM as students, (3) Perceived use of EBM as future clinicians, (4) Barriers to practicing EBM, and (5) Enablers to facilitate the integration of EBM into clinical practice. Key facilitators for promoting EBM to students include competency in EBM, mentorship and application to clinical disciplines. Barriers to EBM implementation include lack of visible application by senior clinicians and constraints by poor resourcing.

**Conclusions:**

The principles and application of EBM is perceived by medical students to be important in both their current clinical training and perceived future work as clinicians. Future research is needed to identify how medical students incorporate EBM concepts into their clinical practice as they gain greater clinical exposure and competence.

## Background

Evidence based medicine (EBM) was introduced as a method for assisting clinicians with obtaining information and synthesising its usefulness to aid clinical decision making. Medical decision making is aided by the principles of EBM when the best available evidence is integrated with a clinician's expertise and patient preferences,[1] regardless of whether the question is one of therapy, diagnosis, harm or prognosis. Elements of evidence based learning and practice include (i) asking an answerable question, (ii) systematically searching for and accessing evidence, (iii) critically appraising the evidence for quality, reliability and robustness, and (iv) integrating useful information into the clinical setting[2].

Many medical schools are teaching EBM as a core requirement in their medical curriculum [3]. The last decade has seen the integration of EBM into the curriculum of medical courses, in both the undergraduate and post-graduate setting, as well as during pre-clinical and clinical years[4-9]. Teaching the principles of EBM to medical students increases knowledge, improves critical appraisal skills and attitudes in both the undergraduate and post-graduate setting[4-9]. Additionally, EBM content can be delivered as a standalone course, or integrated into existing activities within the medical curriculum[2].

The majority of studies to date have focused on the effectiveness of teaching EBM to students in terms of increasing knowledge and skills across varying healthcare disciplines and settings (i.e. undergraduate/post-graduate). Few studies have explored student attitudes, beliefs and uptake of EBM principles and how medical students apply their acquired knowledge and skills in clinical training. A qualitative study of first year medical students identified that EBM and medical statistics were perceived as a static discipline; one that potentially lacks clinical relevance to first year medical students[10]. A lack of clinical maturity may distort the perception that first year medical students have on EBM as an abstract subject, with little clinical relevance; particularly when students are commonly focused on gaining knowledge and skills in other subjects (i.e. anatomy, physiology) that often constitute the core learning in medicine [10].

Perceived student relevance of EBM to clinical practice may increase as students accumulate greater clinical experience throughout their course. A study of third-year American medical students' self perception of their EBM skills reported that the majority believed they incorporated greater EBM principles in their day to day learning experiences, than prior to undertaking the EBM course[11]. A qualitative study of Norwegian undergraduate medical students identified that student attitudes and uptake of EBM principles may be positively modified if educators can demonstrate the link between competency in EBM and its direct influence on study habits and assisting medical decision making in future clinical practice [12]. Not being able to demonstrate this relevance presents as a barrier to promoting EBM in clinical practice. This study also identified that access to appropriate technology and time management may act as a barrier to how students perceive and integrate EBM principles in their daily study [12]. Students are more likely to rely on materials that are not peer-reviewed if access to internet-based medical databases, and journals is problematic [12].

Further evidence is needed to explore student perceptions on EBM and its implementation within the medical curriculum, their use as students and perceived use in the clinical environment. The overall aim of this study was to identify undergraduate medical student perceptions on EBM. The study specifically aimed to explore their current use of EBM principles in their study environment, clinical teaching setting, perceived future use and need of EBM as clinicians and associated barriers and enablers to its use in practice.

## Methods

### Population and Setting

**Figure 1:**
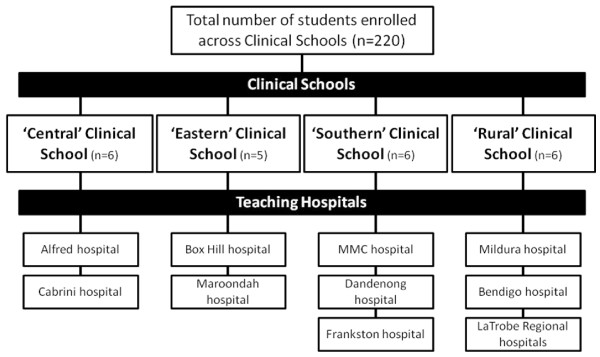
**Structure of the Clinical Schools and associated teaching hospitals within the Monash University MBBS degree**.

This study was set in the context of the medical curriculum at Monash University (Australia). The Monash University medical degree is a five year undergraduate program in which students spend the first two years of their degree on campus. Students commence their clinical training in a hospital setting during the third year of the degree. A total of 220 students were enrolled as third year medical students in 2008, when this study was performed. During this third year students are based for the entire year at the same teaching hospital. Students have the choice of being based at one of seven metropolitan hospitals or five rural hospitals. Each teaching hospital is attached to one of four Clinical Schools (Central, Eastern, Southern or Rural) within the medical degree (Figure 1). Each Clinical School acts as an academic, administrative and clinical intermediary between the University and each respective hospital. Students are provided with an introduction to population health and epidemiology in the first two years of the course. A formal 10-week program in EBM is delivered in the third year of the degree; integrating student's existing knowledge on population health and epidemiology, with principles of EBM in the clinical environment.

The content of the EBM program is detailed in Table 1. The course is delivered in a tutorial format consisting of 12-15 students, with sessions running for two hours. Each session consists of a formal presentation by the tutor, followed by structured small group work and large group discussion. Each tutorial is delivered independently by trained tutors at each of the teaching hospitals. Clinical examples used in the EBM course are derived from existing clinical examples that students encounter when performing their problem based learning (PBL) activities. Additionally, students are required to undertake assessment tasks for the EBM unit, which require students to locate a patient during their clinical rotation, identify a suitable clinical question based on their clinical presentation, source an appropriate article, critically appraise it and state how the results would influence their decision to treat the presenting patient.

**Table 1:** Topics presented in the EBM course

Topic	Content
Introduction to EBM	The principles of EBM are presented and discussed with key examples of EBM in practice
Developing an answerable question	The PICO mnemonic is described, with students practicing writing answerable questions using PICO from a variety of clinical scenarios.
Searching the medical literature (databases including MEDLINE)	Students attend an 'interactive' library session. Students receive a tutorial on how to effectively search MEDLINE, with the tutor demonstrating on a computer projection and students mimicking the search on their computers. Students are provided with clinical scenarios to practice constructing answerable questions and searching for relevant articles on MEDLINE in the remaining time of the tutorial.
Study designs	Content is delivered on how the following study designs are constructed;
	• Randomised controlled trials
	• Cohort studies
	• Case-control studies
	• Systematic reviews
	Specific strengths and limitations of the above study designs are presented and discussed. Also included are methods of bias and overcoming bias in studies (e.g. selection, performance, attrition and detection bias).
Critical appraisal techniques	Critical appraisal techniques for the following clinical questions and study designs are demonstrated;
	• Therapy
	• Harm
	• Diagnosis
	• Prognosis
	• Systematic reviews
	Students are presented with a worked example demonstrating the critical appraisal of an article. Students are then required to perform a critical appraisal of another article in small groups and present answers in a large group discussion at the conclusion of the tutorial.
Biostatistics	Presentations on how to calculate and interpret the following biostatistics are provided;
	• Measures of outcomes (including relative risk, relative risk reduction/increase, absolute risk, absolute risk reduction/increase, number needed to treat and odds ratios)
	• Confidence intervals & p-values
	• Sensitivity, specificity, positive and negative predictive values & likelihood ratios

### Methodological approach

Qualitative research methodology, in the form of focus groups, was used to identify student perceptions regarding EBM. Focus groups were conducted in the week following the conclusion of the 10-week EBM program in 2008. Focus groups were guided by a grounded theory approach, which allowed participants to interact and provide direct opinions and experiences on the issue[13,14]. The use of focus groups was deemed the best methodological approach, since the topic was non-sensitive in nature, whilst the group dynamics could facilitate a robust discussion between participants[15]. The facilitator was guided by an interview schedule, which was developed by results from a survey of the students prior to the focus group discussions. This approach provides a form of methodological triangulation, which provides greater rigor to the study.

### Participants

Third year medical students, who had completed the EBM course were recruited to participate in this study. All students were recruited through a bulk email sent through each clinical teaching hospital. Students interested in participating in the study were asked to respond via email to the course coordinator (DI), from which a suitable time and date for the focus group was formulated. Students were eligible to participate in the study if they were currently enrolled as a third year Monash MBBS student and provided informed consent. Ethics approval for this study was received by the Monash University Standing Committee on Ethics in Research Involving Humans.

### Format

Each focus group was comprised of five to six students who were taught under the same Clinical School, but different teaching hospitals. This approach ensured that each Clinical School was represented by one focus group, with students in that focus group also representing views from the associated teaching hospitals (Figure 1). All participants volunteered to participate in the study and therefore were not paid for their contribution. All focus groups were moderated by the same facilitator (DI), an experienced facilitator but also the coordinator of the EBM unit.

### Data analysis

All focus group discussions were audio taped with a digital recorder and downloaded onto computer for storage. All focus group recordings were transcribed verbatim by an independent company, outsourced specifically for this purpose. All quotes presented are literal transcriptions of the discussions in the focus groups. All transcripts were de-identified, thereby keeping the anonymity of the participants. Focus groups were conducted until the data reached a point of theoretical saturation;[13,14] that is no new themes were generated by further discussion on the topic. Preliminary analysis of transcripts was conducted following each focus group before another commencing another focus group. This approach allows the identification of themes, which may identify new questions for upcoming discussions, or indicate a point of theoretical saturation, whereby following focus group discussions will not result in the generation of new themes. Both investigators decided after the fourth focus group that proceeding with further focus groups would not generate discussion on any new themes.

All transcripts were independently analysed by the two investigators using thematic analysis,[13,14] with the assistance of the NVivo program. This approach was implemented to avoid any potential for bias or pre-existing viewpoints that the lead investigator might have had in his role of course coordinator. Using thematic analysis both investigators independently coded and categorized themes within the data. Common themes were discussed between the two investigators before the data analysis was finalised. Themes were identified using 'group to group validation', whereby each theme was identified by the number of groups mentioning it, the amount of people within each group mentioning it and the overall enthusiasm generated by the theme across the groups [16].

## Results

A total of 23 students, from an eligible population of 220 (10.5%), participated in focus group discussions (Figure 1). Four focus groups were performed, with each focus group representing one of the four Clinical Schools (Central, Eastern, Southern and Rural). Each focus group also had at least one participant from a teaching hospital from the associated Clinical School (i.e. the Southern focus group had 2 representatives each from MMC, Dandenong and Frankston hospitals). This sampling approach ensured that all teaching hospitals and Clinical Schools were represented by at least one participant at each focus group.

Analysis of the audio transcripts from the focus group discussions identified several emerging themes (Table 2). Of the identified themes, five were consistently present across all focus groups. These five themes were considered as the 'major' themes, with results presented below. Three themes were deemed to be 'minor' as they were not consistently identified across all focus groups. These minor themes are listed in Table 2.

**Table 2:** Themes identified from focus group discussions

Theme	Focus group (Clinical School site)
**Major Themes**	
1. Rationale and observed use of EBM in practice	Central, Southern, Eastern, Rural
2. Use of EBM principles as students	Central, Southern, Eastern, Rural
3. Perceived use of EBM as future clinicians	Central, Southern, Eastern, Rural
4. Barriers to practicing EBM	Central, Southern, Eastern, Rural
5. Enablers to practicing EBM	Central, Southern, Eastern, Rural

**Minor Themes**	
6. Critical appraisal techniques	Central, Southern
7. Implementing research into practice	Rural, Southern
8. Searching the medical literature	Eastern, Rural

### Summary of Major Themes

#### 1. Rationale and observed use of EBM in practice

The majority of students defined EBM as the classic definition of integrating evidence with medical decision making. EBM was defined as a construct on which clinicians may make medical decisions based on the best current evidence. Most of the students perceived the need for integrating evidence in their daily practice, both currently as students and in the future as practicing clinicians.

'(EBM) is practising things that have evidence behind them, rather than just assuming that they work.'

Students believed that EBM allows the application of research/evidence into practice to help make the best patient decisions. Students also stated that it provides clinicians with a justification to express alternative options to patients (such as a comparison between two competing treatments), with the guidance of quantified measures of effectiveness (statistics) and the degree to which these may vary due to different factors.

'You can give patients options as well, I guess you could say well this one is there to show this and this one's there to show that...(in reference to drug treatments).'

Students identified that the use of EBM was variable across their experience within the medical curriculum and specifically in their clinical teaching environment. Whilst students believed that EBM could be incorporated across all fields of medicine, they observed that its use was often discipline specific. Students reported the integrated use of EBM principles by clinicians in practice and during bedside teaching activities when undertaking rotations in emergency medicine, general medicine and cardiology; often by early to mid-career clinicians.

'...you often hear lots of the registrars and the physician trainees all talking about like what the recent studies have been and they seem to apply a lot to...'

#### 2. Use of EBM principles as students

Students stated that the Monash EBM program provided students with an understanding of fundamental knowledge and skills which were immediately applicable across a variety of clinical settings. Information gained through the EBM course was perceived to be timely and relevant as it was integrated with their first clinical experience in the hospital environment.

'(Undertaking the EBM program) you think more about why you're doing the test rather than just thinking to do it (during clinical rotations). You think well, that will give me this sort of information versus this sort of information.'

Students discussed the advantage of applying specific EBM skills in assisting their study behaviour. The ability to search, appraise and integrate evidence into tasks such as Problem Based Learning (PBL) scenarios was highly regarded. Students stated that the skills learnt in effectively searching the medical literature, provided greater scope to efficiently search medical databases to obtain relevant information and directly apply it to better understand therapeutic interventions or diagnostic test results.

'The biggest use I find for Medline is finding articles on specific topics, like how to read an x-ray, how to read a chest x-ray.'

'I was doing my task today. It was comparing mastectomy to conservative surgery for breast cancer. I used 'Up To Date', which gave me all the latest information. It gave me all the studies that had been done, which was useful.'

However, the actual application of this process was principally determined by the expectations placed on them by clinical supervisors. Students with tutors who were interested in EBM perceived a greater expectation to apply EBM principles when completing their PBLs, whilst other groups with tutors not proficient in EBM did not experience this behaviour.

#### 3. Perceived use of EBM as future clinicians

Students acknowledged that they were clinically inexperienced and not able to currently make decisions with patients. However, they expected their use of EBM skills to increase as they began their internship and had the opportunity to influence patient outcomes. All students believed that they would base their practice as clinicians on evidence based approaches to medicine.

'I think it's surprising to think that you'd do it (practice medicine) any other way really. It's so rational of course; you should use things that have been proven'

Students believed that they will continue to use EBM throughout their studies and throughout their clinical careers. This view stemmed from the belief that their competence in EBM will provide them with skills to keep abreast of the most current evidence in a medical environment that is perceived to be constantly changing. It was also suggested that being taught EBM principles in the undergraduate environment would provide them with the knowledge and skills to facilitate continued uptake of knowledge and professional development throughout their careers as students and clinicians.

'By the time you're completed your degree you're expected not to feign interest in EBM, but actually base your teaching and practice on good solid evidence based medicine.'

Students believed that it would be extremely difficult to incorporate EBM principles on a daily basis, for example continually searching the literature and appraising studies to apply to clinical scenarios. However, they reasoned that they now had the necessary expertise to identify relevant literature, appraise its usefulness and implement the evidence according to their perceived need in the clinical situation. They perceived that they now had the ability to critically appraise literature and critically think about significance and applicability of interventions in practice.

'I don't know that I'd specifically use it (critical appraisal) but once you know it you always think about it when you're looking at things... I probably would never go and do specific re-calculations of the data, but I might wonder if that result really is significant.'

'I probably would never go and do any calculations [for sensitivity/specificity], but like you just think oh I know what that means, if that test result is significant or whatever, so it's useful in that regard.'

#### 4. Barriers to practicing EBM

Students identified that the key barrier to the implementation of EBM principles in practice was changing the behaviour and current practice of older, senior clinicians. Students hypothesized that few senior clinicians would modify their current approach to practicing medicine based on what they perceived as 'negligible' findings from studies. Students suggested that the incorporation of technology may be a primary barrier for such older clinicians wishing to keep abreast with the current evidence but not possessing the necessary computer skills to do so (e.g. searching Medline and retrieving articles efficiently).

'There's some things that no matter what the evidence is, the consultant has to learn a whole new way of doing things, and he's not going to do that.'

'It's individual... some of them (consultants) are open to the new things, you know, evidence based medicine but then there's other things where they'd rather just stick to the old things, what they're used to.'

Students recognized the importance of practicing EBM, and expressed concern for clinicians who seemingly didn't adopt this practice. Many students viewed that incorporating the current EBM course into the medical training curriculum as a method of overcome this barrier in future generations of clinicians. However, it was acknowledged that it would take a 'generational' change to see principles of EBM integrated across early, mid and experienced clinicians.

'It's hard to imagine a world where evidence based medicine didn't exist and they just did it willy nilly.'

#### 5. Enablers to practicing EBM

Students described that observing their clinical supervisors referring to research and evidence when discussing patient cases with other senior clinicians as the leading enabler for undertaking and implementing and utilising EBM themselves. Furthermore, undertaking the EBM unit was perceived as providing a solid grounding with which they could relate to their mentors and actively participate in learning activities.

'We're able to understand when clinicians refer to journals and things ... when they say there's evidence for this and that and this is why we're doing this, we understand.'

Students perceived that more early and mid career clinicians tended to adopt an EBM approach to their practice. The majority of these clinicians were also their clinical tutors, therefore they also influenced the manner in which students observed and appreciated the need for EBM in practice. Students noted that observing clinicians practicing EBM provided them with the impetus to also do so.

'It's embedded in the culture, like I see everybody doing it so I think I'll do it".'

Students believed that the greatest enabler for promoting EBM in practice was the autonomy it gives to clinicians in continuing further education and keeping up to date with current evidence in medicine. They recognised that as clinicians greater emphasis that will be placed on maintaining current knowledge in their chosen discipline and that the knowledge and skills gained through the EBM program will facilitate its uptake.

'It is expected that you will remain up to date in your chosen field... you're expected to base your teaching and your practice on good solid evidence based medicine.'

### Summary of Minor Themes

Whilst not present across all focus groups, several minor themes were also identified. These themes (Critical Appraisal Techniques, Implementing Research into Practice and Searching the Medical Literature) represented students' self perceptions of the skills component associated with EBM. Students perceived that their skills in searching for medical literature across different databases improved throughout the course.

"There was a PBL (problem based learning activity) we had to do on gender differences in presentation for AMI (acute myocardial infarction); both Medline and Up To Date were really useful in finding relevant information."

Students also identified critical appraisal as an important component in evaluating medical information; particularly as its implementation may change existing treatment or diagnostic regimes.

" It (critically appraising an article) might show there was a huge benefit, so then it would make a difference and you'd use it (an intervention). But when there's just a minor difference (between existing treatments and new treatments), you'd just keep using the old treatment."

## Discussion

The principles and application of EBM is perceived by medical students to be important in both their current clinical training and perceived future work as clinicians. Facilitators for promoting EBM within study and future clinical work include competency in EBM principles, mentorship from clinicians adopting an evidence-based approach to clinical practice, and a perceived relevancy for implementing the EBM approach within clinical practice. Barriers to EBM implementation include disengagement between senior clinicians and EBM, lack of implementation with specific medicine disciplines and impact of available resources (technology and time).

Study participants supported the view that medical students will be more likely to adopt EBM principles in their learning and clinical experience when there is a demonstrable link between classroom teachings of EBM and clinical application[12]. Unlike first-year medical students, who get no clinical contact, these third-year medical students are experiencing their first opportunity to practice what they have been taught thus far in their degree. Contrary to first-year medical students who may lack clinical maturity, [10] participants in this study demonstrated that third-year medical students can appreciate the clinical relevance and the fundamental approach to EBM in linking evidence with the clinical experience and patient expectations.

Medical students being taught the principles of EBM in an integrated manner within the curriculum perceive the need to adopt evidence-based learning as students, and practice as clinicians [11]. Teaching EBM to medical students in an environment in which they can directly apply their EBM knowledge and skills in daily practice, and have that practice reinforced by clinicians who support the use of an evidence-based approach to practice, may prolong their preference to actively continue to practice according to the principles of EBM.

Students in this study identified a dichotomy regarding barriers and enablers to the use of EBM principles in practice. Some clinicians may actively promote the use of EBM, whilst others may not emphasise its use as much and tend to rely more on their clinical expertise. Clinicians who do not actively support the use of EBM principles in practice may do so for a variety of reasons including lack of time, resources, skills or belief in the net worth of EBM [17,18]. Regardless of attitudes either for, or against, EBM; medical students and clinicians alike must appreciated that any evidence should assist, rather than displace a clinicians' expertise and experience [19].

### Limitations

Volunteer sampling was used to recruit third year medical students to the study. This approach may introduce a degree of selection bias to the study; as such volunteers may have attitudes and perceptions of EBM that may not necessarily be representative of the entire year cohort. Similarly, it was beyond the scope of this study to obtain data on reasons as to why other students chose not to participate in focus group discussions. The use of the course coordinator as the facilitator of the focus groups had the potential to prohibit students from critiquing aspects of the course and the topic that they may have otherwise done with a facilitator not associated with the course, Faculty or University. Despite this potential for bias, students commented during the focus groups that the presence of the course coordinator did not influence their responses. All tutors have training in the principles of EBM however; it was not possible to determine the level of expertise of each tutor in EBM or educational training. Greater expertise and application of EBM in clinical practice by tutors may influence the manner in which the EBM materials can be presented to students, which may affect the manner in which students perceived EBM.

### Future research and implications

This study explored third-year medical students' perceptions of EBM, the first year in which they are exposed to clinical work and based at a hospital for an entire year. Future research should explore how these perceptions change, if at all, during the remaining time of their clinical education. For example, fourth-year medical students within the Monash University MBBS degree undertake four nine-week rotations across various disciplines, including general practice, psychiatry, obstetrics and gynaecology. There is a dearth of information exploring how EBM is perceived in such disciplines, and what effect, if any; such a culture may have upon undergraduate medical students. Similarly, interviews with clinicians would provide an insight into how they view EBM and how their prejudices, either for or against EBM, influence the manner in which they teach medical students. Further work, incorporating a mixed-methods approach, is also needed to further generalise the results of this research to other populations. A cross-comparison study investigating differences between medical, nursing and other allied health disciplines would be warranted.

## Conclusions

EBM has been described as an essential way of teaching and practicing medicine in an uncertain environment[2]. Using an evidence-based approach to practice medicine is much desired, but often faces practical and cultural barriers. Mentorship from senior clinicians, and a good understanding of the practical benefits and limitations associated with EBM may promote an environment in which students may adopt EBM principles as students, as well as embedding it in their practice as future clinicians.

## Competing interests

DI is the coordinator of the EBM program for the MBBS degree at Monash University.

## Authors' contributions

DI designed the study, collected the data, performed the secondary data analysis and drafted the manuscript. KF participated in data collection, performed the primary data analysis and helped to draft the manuscript. All authors read and approved the final manuscript.

## Author information

DI is a Senior Lecturer in Evidence Based Clinical Practice at the School of Public Health & Preventive Medicine, Monash University.

KF is a Research Assistant at the School of Public Health & Preventive Medicine, Monash University.

## Pre-publication history

The pre-publication history for this paper can be accessed here:

http://www.biomedcentral.com/1472-6920/10/58/prepub
